# Case Report: Intrahepatic bile duct injury during adhesiolysis mimicking anastomotic leak

**DOI:** 10.3389/fsurg.2026.1838302

**Published:** 2026-07-10

**Authors:** Yunchul Park, Young-Goun Jo, Hyunseok Jang, Naa Lee, Euisung Jeong, Hyunseok Roh, Hyo-Sin Kim

**Affiliations:** Department of Surgery, Chonnam National University Medical School and Hospital, Gwangju, Republic of Korea

**Keywords:** adhesiolysis, bile leak, case report, gastrointestinal surgery, intrahepatic bile duct injury, trauma

## Abstract

Intrahepatic bile duct injury (IHBDI) is a rare but serious postoperative complication, typically associated with hepatobiliary procedures or direct hepatic trauma. We present the case of a 79-year-old male with prior distal gastrectomy and Billroth II reconstruction who underwent emergency gastrointestinal surgery following blunt abdominal trauma. Postoperatively, gross bilious output from surgical drains raised concern for anastomotic leakage; however, re-laparotomy revealed bile extravasation from the segment 3 liver surface, consistent with peripheral IHBDI most plausibly arising from a combination of pre-existing peripheral biliary dilatation, occult traumatic injury, and the dissection forces applied during perihepatic adhesiolysis. The injury was successfully managed with direct suture ligation, leading to immediate resolution of bile leakage and complete recovery. This case underscores the diagnostic challenge IHBDI poses in non-hepatobiliary surgery and emphasizes the importance of considering peripheral bile duct injury when unexplained postoperative bile leakage occurs in patients with prior abdominal surgeries.

## Introduction

Bile leak can occur in various clinical contexts, including trauma, hepatobiliary surgery, inflammation, or iatrogenic injury (1, 2). Its clinical manifestations are often nonspecific—such as abdominal discomfort, bilious drainage, or biochemical abnormalities—necessitating imaging studies and correlation with the patient's surgical or clinical background for accurate diagnosis.

In patients with a history of gastrointestinal surgery, postoperative bilious drainage typically raises concern for anastomotic leakage as the primary diagnosis. However, this exclusive focus may result in overlooking other potential sources of bile leakage that require different management approaches. Intrahepatic bile duct injuries (IHBDI) most commonly occur with high-grade liver trauma or as complications of hepatic surgery, with reported incidences of 0.4%–8% after hepatic resection and up to 20% after high-grade blunt hepatic trauma (3, 4). In contrast, IHBDI complicating non-hepatobiliary abdominal surgery—where injury arises from indirect mechanisms such as perihepatic adhesiolysis rather than intentional transection of liver parenchyma—is exceptionally rare, with only isolated case reports describing iatrogenic intrahepatic ductal injury during such procedures (2, 5). Clinicians should therefore maintain vigilance even in less obvious scenarios, as delayed diagnosis can significantly impact outcomes.

What makes this case unique is the occurrence of IHBDI as a complication of perihepatic adhesiolysis rather than of intentional hepatic resection or parenchymal transection. Dense perihepatic adhesions—formed from prior Billroth II reconstruction—were dissected directly off the liver capsule, and disruption of these capsular and subcapsular planes ultimately compromised a peripheral intrahepatic bile duct, producing a leak that initially mimicked anastomotic dehiscence. To our knowledge, this specific mechanism is rarely documented in the literature and represents an important clinical pitfall.

## Case description

A 79-year-old male presented following a motor vehicle collision with blunt abdominal trauma. His medical history was significant for distal gastrectomy with Billroth II reconstruction performed approximately 15 years earlier; he had no prior hepatobiliary procedures and no known liver or biliary disease. Family history was non-contributory for hepatobiliary or oncologic disease, and his social and psychosocial history was likewise non-contributory to the present illness. No genetic testing was performed, as no hereditary condition was suspected. Upon admission, physical examination revealed signs of peritonitis. Computed tomography (CT) demonstrated small bowel perforation with a blow-out defect requiring urgent surgical intervention. Initial laboratory values were notable for leukocyte count 4.5 × 10^3^/μL, hemoglobin 12.1 g/dL, platelet count 203 × 10^3^/μL, serum albumin 3.3 g/dL, aspartate aminotransferase 20 U/L, alanine aminotransferase 14 U/L, total bilirubin 0.82 mg/dL, blood urea nitrogen 18.1 mg/dL, and serum creatinine 0.92 mg/dL. C-reactive protein was below detectable limits (<0.01 mg/dL). Axial and coronal CT images demonstrated peripheral biliary dilatation, suggestive of possible biliary tract involvement (Figure 1).

**Figure 1:**
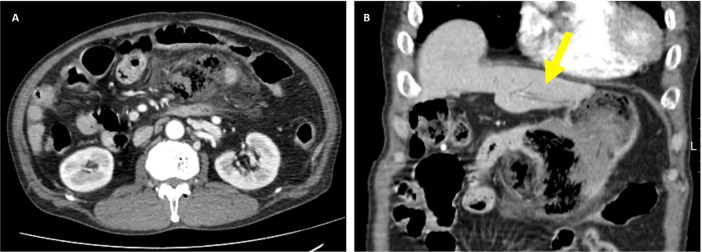
Preoperative abdominal CT scan obtained during evaluation for blunt abdominal trauma. **(A)** Axial image at the level of the porta hepatis. **(B)** Coronal reformatted image; the yellow arrow indicates mild peripheral biliary dilatation in the left hepatic lobe (segment 3), noted incidentally on the trauma protocol scan. The pre-existing dilatation may reflect age-related ductal ectasia, chronic enteric reflux–associated ascending cholangiopathy after remote Billroth II reconstruction, or acute trauma-related periductal edema, and may have contributed to the biomechanical vulnerability of the peripheral duct during subsequent adhesiolysis.

Emergent exploratory laparotomy revealed dense perihepatic adhesions, perforation at the gastrojejunal anastomosis, and a perisplenic hematoma. Surgical intervention included small bowel resection, partial gastrectomy with gastrojejunostomy reconstruction, adhesiolysis, and omentectomy. During adhesiolysis near the liver, sharp dissection was used preferentially, with judicious low-power monopolar electrocautery applied only for focal hemostasis when sharp dissection alone proved insufficient in the densest adhesion planes. On completion of adhesiolysis, the hepatic surface was systematically inspected; no gross bile staining, capsular tear, or parenchymal disruption was identified at that time, consistent with the occult nature of the injury that became apparent only postoperatively. Multiple drains were placed in the subhepatic space, splenic bed, and pelvis.

The dense perihepatic adhesions encountered intraoperatively were consistent with the direct sequelae of the remote Billroth II reconstruction.

### Diagnostic assessment, therapeutic intervention, follow-up and outcomes

#### Diagnostic assessment

On postoperative day one, the appearance of gross bilious output from the surgically placed drains became the principal trigger for clinical concern, accompanied by deteriorating hemodynamic status and worsening systemic inflammatory parameters (rising leukocytosis, anemia, and hypoalbuminemia). Liver function tests showed only mild changes (AST 78 U/L, ALT 38 U/L) that are common after major trauma and emergency laparotomy and were therefore considered nonspecific; the clinical decision was driven by the gross bilious effluent and hemodynamic trajectory rather than by transaminase values. Given the patient's recent gastrointestinal reconstruction, anastomotic dehiscence was initially suspected as the most likely etiology. Additional imaging modalities such as MRCP or hepatobiliary scintigraphy, although potentially useful for source localization in the elective setting, were not pursued because (i) the patient remained intubated and hemodynamically unstable in the intensive care unit, making transfer for advanced imaging impractical, (ii) the bilious drain output already established the presence of a bile leak, and (iii) intraoperative re-exploration was judged to be both more rapid and directly therapeutic. Immediate re-exploration was therefore performed; all intestinal anastomoses were confirmed intact, and bile staining was observed over segment 3 of the left hepatic lobe. Gentle compression of this region elicited active bile extravasation from the liver surface, consistent with peripheral intrahepatic bile duct injury (Figure 2A). From a prognostic standpoint, peripheral IHBDI involving an isolated subsegmental duct is generally amenable to direct surgical repair with favorable long-term outcomes; the principal late risk is delayed biliary stricture, which was prospectively monitored by liver function testing and follow-up imaging.

**Figure 2:**
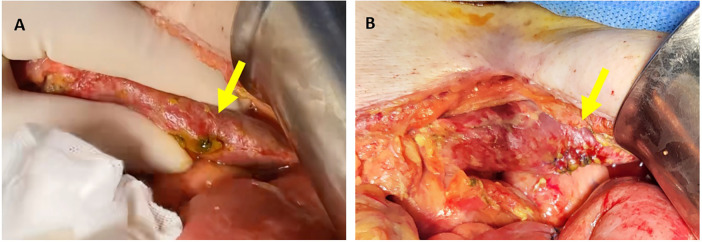
Intraoperative findings confirming intrahepatic bile duct injury. **(A)** Bile staining over segment 3 of the liver surface with active bile leakage identified during re-exploration (arrow). **(B)** Successful suture ligation of the injured peripheral intrahepatic bile ducts using 5-0 prolene, with resolution of leakage confirmed intraoperatively.

#### Therapeutic intervention

Definitive management consisted of direct suture ligation of the injured peripheral bile ducts using 5-0 prolene. Following repair, the integrity of the closure and the absence of additional leakage points were confirmed by gentle saline irrigation of the segment 3 surface together with sustained direct intraoperative visual inspection; no further bile extravasation was observed (Figure 2B). Formal intraoperative cholangiography was not performed because the injury was confined to a peripheral subsegmental duct on the inferior surface of segment 3, well distal to any major biliary confluence, making clinically significant downstream obstruction biomechanically unlikely; the patient's hemodynamic instability also weighed against additional operative time. No biliary stenting or endoscopic intervention was required. Given the immediate and sustained intraoperative resolution of the leak, no further or alternative therapeutic interventions—such as endoscopic biliary stenting, percutaneous transhepatic drainage, or hepaticojejunostomy reconstruction—were undertaken; the therapeutic plan did not require modification in the postoperative course.

#### Follow-up and outcomes

Bilious drainage completely resolved immediately following the second surgery. Hepatic function tests normalized by postoperative day seven. Enteral nutrition was gradually re-established, and the patient was discharged in stable condition. At three-month follow-up, the patient remained asymptomatic with normal liver function tests and no evidence of biliary stricture on follow-up imaging.

## Discussion

The patient's clinical course is summarized in Table 1. This case illustrates an unusual circumstance in which a segmental intrahepatic bile duct sustained injury as a complication of perihepatic adhesiolysis rather than of intentional hepatic parenchymal resection. We deliberately avoid the framing of injury occurring “without hepatic manipulation”: dissecting dense adhesions directly off the liver capsule constitutes, by definition, manipulation of the perihepatic and subcapsular environment, and the present injury should be understood in that light. Rather than a single isolated mechanism, the injury most plausibly reflects the convergence of several contributing factors. First, the preoperative CT demonstrated pre-existing mild peripheral biliary dilatation in the left lobe; whether this represented age-related ductal ectasia, chronic enteric reflux–associated ascending cholangiopathy following the remote Billroth II reconstruction, or acute trauma-induced periductal edema cannot be determined retrospectively, but a dilated and potentially thinned peripheral duct is biomechanically more vulnerable to shear and traction forces than a normal-caliber duct. Second, the patient's high-energy motor vehicle collision is highly likely to have produced an occult contusional injury to the left hepatic surface that was not grossly evident on intraoperative inspection at the index operation; such occult subcapsular injury may have weakened the local parenchyma and ductal wall before adhesiolysis. Third, dissection of dense perihepatic adhesions formed from the prior Billroth II reconstruction—performed predominantly with sharp technique but supplemented by focal low-power monopolar electrocautery—imposed traction, shear, and probable focal thermal stress on the already vulnerable subcapsular tissue. The exact contribution of each component cannot be determined from a single case, but the most parsimonious explanation is a “two-hit” or multifactorial mechanism: a duct rendered fragile by pre-existing dilatation and occult blunt trauma was then disrupted during subsequent capsular and subcapsular dissection. This integrated framework is more consistent with the operative findings than attributing the injury to any single isolated cause.

**Table 1:** Timeline.

Time Point	Event	Clinical findings/Management
Presentation	Blunt abdominal trauma (MVA)	Leukocytosis, normal LFTs; CT: small bowel perforation, mild peripheral biliary dilatation
Emergency surgery (Day 0)	Exploratory laparotomy	Small bowel resection, partial gastrectomy, gastrojejunostomy reconstruction, adhesiolysis; drains placed in subhepatic space
POD 1	Bilious drain output	Worsening leukocytosis, rising AST/ALT (78/38 U/L); clinical suspicion of anastomotic leakage → re-laparotomy
Re-laparotomy (POD 1)	IHBDI identified	All anastomoses intact; bile staining over segment 3; active leakage confirmed by compression → suture ligation with 5-0 prolene
POD 1–7	Post-repair recovery	Complete resolution of bilious drainage; LFTs normalized by POD 7
Discharge	Stable condition	Enteral nutrition re-established; discharged in good condition
3-month follow-up	Outpatient review	Asymptomatic; normal LFTs; no biliary stricture on follow-up imaging

MVA, motor vehicle accident; POD, postoperative day; LFT, liver function test; IHBDI, intrahepatic bile duct injury; AST, aspartate aminotransferase; ALT, alanine aminotransferase.

Bile leak most frequently complicates laparoscopic cholecystectomy (6), hepatic resection, and ERCP. Additional reported etiologies include blunt hepatic trauma, radiofrequency ablation, and hepatic abscess formation (1, 2). IHBD injury typically occurs with hepatectomy, liver trauma, or radiofrequency ablation (2, 4). A similar case of bile leakage during right hemicolectomy was reported by Lee et al. (5), which most closely resembles the mechanism described here—iatrogenic IHBDI without direct hepatic surgery. Our case adds to this rare body of literature by documenting the same phenomenon following adhesiolysis in a trauma setting. Beyond anastomotic dehiscence, the differential diagnosis of postoperative bilious drainage after upper gastrointestinal surgery should also include cystic duct stump leak, injury to the extrahepatic biliary tree, duodenal stump dehiscence, gallbladder fossa leak following unrecognized cholecystectomy-related injury, and—as illustrated here—peripheral IHBDI arising from adhesiolysis.

Initial misidentification of the bile source as anastomotic leakage represents the critical diagnostic challenge in this case and in similar presentations. Diagnostic tools including CT, MRCP, and HIDA scan can be utilized to evaluate bile leak (7–9); however, CT alone may be insufficient to confirm the diagnosis in complex clinical settings. Bilious drainage from surgically placed drains can identify bile leak, but determining the underlying source remains challenging (10). The key lesson is that when bilious drainage occurs after surgery involving adhesiolysis near the liver—particularly in patients with prior upper abdominal procedures—peripheral intrahepatic bile duct injury should be included in the differential diagnosis even when no hepatic resection has been performed.

Management of bile leak depends on leakage volume, source, and the patient's condition. Many cases are effectively managed with percutaneous drainage, ERCP, or biliary stent insertion (1). When conservative approaches fail, surgical intervention is necessary. In our patient, direct suture ligation provided definitive control with an excellent outcome. Importantly, had anastomotic leakage been incorrectly assumed and the hepatic source overlooked, the patient may have developed life-threatening sepsis. Timely intraoperative identification was therefore critical.

### Strengths and limitations

The main strength of this report is the clear demonstration of a mechanistically distinct cause of postoperative bile leak, with well-documented intraoperative findings and complete follow-up. Several limitations must be acknowledged. First, this is a single case report, limiting generalizability. Second, the exact mechanism of injury could not be definitively established at the time of operation; pre-existing peripheral biliary dilatation, occult contusional injury from the antecedent motor vehicle collision, and the dissection forces (sharp and focal thermal) applied during adhesiolysis are all plausible contributors, and their relative weights cannot be resolved retrospectively. Third, the etiology of the pre-existing peripheral biliary dilatation itself remains uncertain; choledocholithiasis was not demonstrated on preoperative imaging, and the differential includes age-related ductal ectasia, enteric reflux–associated ascending cholangiopathy related to the prior Billroth II reconstruction, and acute trauma-induced periductal edema. Fourth, formal intraoperative cholangiography and postoperative MRCP were not obtained after suture ligation; downstream patency was instead inferred from the peripheral subsegmental location of the injury (well distal to any major biliary confluence), the absence of further extravasation on intraoperative saline irrigation and direct inspection, the immediate and sustained resolution of bilious drain output, the rapid normalization of liver function tests, and the absence of stricture or ductal abnormality on three-month follow-up imaging. Although the favorable clinical course supports the adequacy of the repair, we acknowledge that formal cholangiographic confirmation would have strengthened the intraoperative assessment, and routine intraoperative cholangiography may be reasonable when peripheral IHBDI is identified and suture-ligated in less anatomically favorable locations.

### Take-away lessons

Surgeons performing adhesiolysis near the liver, particularly in patients with complex prior upper abdominal surgery or recent blunt abdominal trauma, should maintain heightened vigilance for peripheral intrahepatic bile duct injury. When unexplained postoperative bile leakage occurs, the source should not be assumed to be anastomotic without thorough intraoperative re-exploration. Practical preventive measures, consistent with our operative experience, include: maintaining the dissection plane on the adhesion side rather than on the liver capsule whenever feasible; controlled traction–countertraction to minimize shear on the capsular surface; restricting monopolar electrocautery to focal, low-power, brief applications and preferring bipolar energy or sharp division near the liver capsule to limit collateral thermal injury to subcapsular bile ducts; and systematic visual inspection of the entire mobilized hepatic surface on completion of adhesiolysis, with consideration of saline irrigation testing in high-risk areas. Intraoperative cholangiography or postoperative MRCP should be considered when occult bile duct injury is suspected, especially if the injured duct lies near a major biliary confluence. Recent evidence emphasizing complexity-based risk stratification for postoperative complications after hepatobiliary procedures further supports vigilant early postoperative monitoring, particularly in patients with complex prior abdominal surgery (11). Early surgical identification and direct suture ligation of peripheral IHBDI can result in excellent outcomes and prevent further morbidity.

## Patient perspective

The patient expressed appreciation for the prompt surgical response when his condition changed unexpectedly after the initial operation. He reported being particularly relieved that the source of the problem was identified and repaired quickly. At his three-month follow-up, he stated that he had returned to his baseline activities of daily living and experienced no ongoing abdominal discomfort or jaundice. He provided written informed consent for publication of this case report.

## Data Availability

The raw data supporting the conclusions of this article will be made available by the authors, without undue reservation.
